# Dysregulation of the Autophagy-Endolysosomal System in Amyotrophic Lateral Sclerosis and Related Motor Neuron Diseases

**DOI:** 10.1155/2012/498428

**Published:** 2012-07-17

**Authors:** Asako Otomo, Lei Pan, Shinji Hadano

**Affiliations:** ^1^Department of Molecular Life Sciences, Tokai University School of Medicine, 143 Shimokasuya, Isehara 259-1193, Japan; ^2^The Institute of Medical Sciences, Tokai University, 143 Shimokasuya, Isehara 259-1193, Japan; ^3^Research Center for Brain and Nervous Diseases, Tokai University Graduate School of Medicine, 143 Shimokasuya, Isehara 259-1193, Japan

## Abstract

Amyotrophic lateral sclerosis (ALS) is a heterogeneous group of incurable motor neuron diseases (MNDs) characterized by a selective loss of upper and lower motor neurons in the brain and spinal cord. Most cases of ALS are sporadic, while approximately 5–10% cases are familial. More than 16 causative genes for ALS/MNDs have been identified and their underlying pathogenesis, including oxidative stress, endoplasmic reticulum stress, excitotoxicity, mitochondrial dysfunction, neural inflammation, protein misfolding and accumulation, dysfunctional intracellular trafficking, abnormal RNA processing, and noncell-autonomous damage, has begun to emerge. It is currently believed that a complex interplay of multiple toxicity pathways is implicated in disease onset and progression. Among such mechanisms, ones that are associated with disturbances of protein homeostasis, the ubiquitin-proteasome system and autophagy, have recently been highlighted. Although it remains to be determined whether disease-associated protein aggregates have a toxic or protective role in the pathogenesis, the formation of them results from the imbalance between generation and degradation of misfolded proteins within neuronal cells. In this paper, we focus on the autophagy-lysosomal and endocytic degradation systems and implication of their dysfunction to the pathogenesis of ALS/MNDs. The autophagy-endolysosomal pathway could be a major target for the development of therapeutic agents for ALS/MNDs.

## 1. Introduction

Amyotrophic lateral sclerosis (ALS) is a heterogeneous group of inexorable neurodegenerative disorders characterized by a selective loss of upper and lower motor neurons in the brain and spinal cord [1, 2]. Most patients die of respiratory failure within 3–5 years. Although ALS is one of the best studied and a well-known form of motor neuron diseases (MNDs), the molecular pathogenesis of ALS is still unclear [1, 2]. To date, no effective therapeutic interventions to cure or even relieve symptoms are available [3].

Most cases of ALS are sporadic, while approximately 5–10% cases are familial. Recent advances in human genetics and genomics greatly facilitate the chromosomal mapping of disease loci, and the identification of causative genes and mutations predisposing to many familial forms of ALS/MNDs [1]. Thus far, more than 16 ALS causative genes including *SOD1*, *ALS2*, *SETX*, *SPG11*, *FUS*, *VAPB*, *ANG*, *TARDBP*, *FIG4*, *OPTN*, *ATXN2*, *VCP*, *C9orf72*, *UBQLN2*, *SIGMAR1*, and *CHMP2B* have been identified [1, 4] (http://neuromuscular.wustl.edu/index.html) (Table 1). The following characterizations of the disease-causing and -related gene products, in conjunction with the creation of animal models, have successfully unveiled the molecular basis underlying the pathogenesis of ALS/MNDs, such as oxidative stress, endoplasmic reticulum (ER) stress, excitotoxicity, mitochondrial dysfunction, neural inflammation, protein misfolding and accumulation, dysfunctional intracellular trafficking, abnormal RNA processing, and noncell-autonomous damage [4–11]. It is currently believed that a complex interplay of such multiple toxicity pathways, rather than a single independent mechanism, is implicated in the ALS/MND's pathogenesis [4–6].

 Among these pathogenic mechanisms, ones that are associated with disturbances of protein homeostasis have been highlighted, as the accumulation of insoluble protein aggregates is the cardinal pathological feature for ALS and other neurodegenerative diseases [12]. Although it remains to be determined as to whether such protein aggregates have a toxic or protective role in the pathogenesis of ALS/MNDs, it is conceivable that the formation of them results from the imbalance between generation and degradation of misfolded proteins within neuronal cells. In eukaryotes, there are two main degradation systems for cytoplasmic proteins, that is, the ubiquitin-proteasome system (UPS) and autophagy. The UPS is mainly involved in selective clearance for short-lived proteins [13], while autophagy is the mechanism by which the long-lived as well as misfolded proteins can be removed by the endolysosomal system [14, 15]. It is also noted that the involvement of endocytosis and vesicle trafficking in the regulation of protein homeostasis and degradation have recently emerged [10, 16–18].

In this paper, we aim to give a comprehensive view on the autophagy-endolysosomal system and implication of its dysfunction to the pathogenesis of ALS/MNDs. Excellent review articles specialized on the role of the UPS in ALS/MNDs can be found elsewhere [13, 19].

## 2. The Autophagy-Endolysosomal System

### 2.1. Autophagic Pathways

Autophagy is an evolutionally conserved lysosomal degradation system that is tightly linked to a wide variety of physiological processes such as protein homeostasis, removal of pathogens, and antigen presentation. There are at least three forms of autophagic pathways; macroautophagy, microautophagy, and chaperon-mediated autophagy, among which macroautophagy, hereafter referred to as “autophagy”, plays a crucial role in the removal of cytoplasmic long-lived as well as misfolded proteins [14, 20–23]. Autophagy comprises three sequential steps; autophagosome formation, maturation, and degradation within lysosomes, through which entrapped cargo molecules within autophagosomes can be degraded and reutilized for the synthesis of newer cellular components.

These multiple steps of autophagy are highly orchestrated by a common group of proteins called ATG (autophagy-related), such as Atg5 and Atg7 [14], and Rab GTPases, a large family of small G proteins [24]. While most autophagic pathways are Atg5/Atg7-dependent, an Atg5/Atg7-independent but Rab9-dependent alternative autophagic pathway has recently been found in mammals [25]. Although autophagy is highly upregulated under stress conditions such as nutritional starvation [14] and exercise [26] (BCL2/Beclin-1-dependent inducible autophagy), several lines of evidence support the existence of basal or constitutive autophagy (BCL2/Beclin-1-independent basal autophagy) in most cell types including neuronal and muscle cells [26–30]. In fact, despite that starvation does not induce autophagy in the brain [31], either Atg5 or Atg7 deficiency in neurons results in the accumulation of misfolded proteins and neurodegeneration [29, 30], indicating that basal autophagy has a crucial role in the central nervous system (CNS).

**Figure 1:**
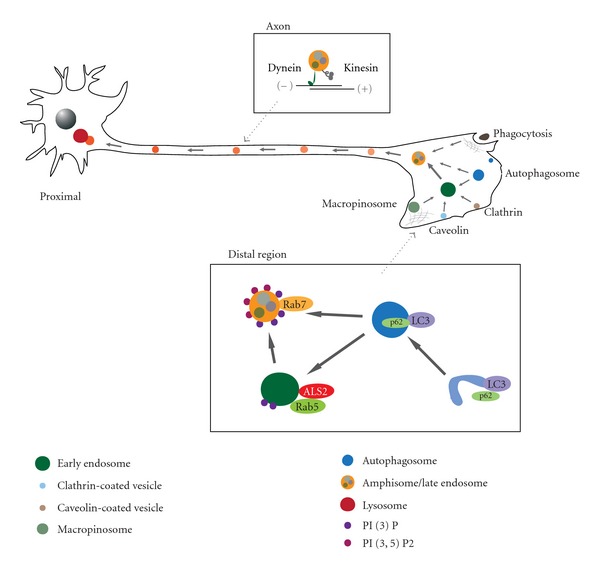
Endocytic trafficking and the autophagy-endolysosomal system in neurons.

Whichever autophagic mechanisms except Atg5/Atg7-independent alternative [25] are involved, their activation can be monitored by the level and distribution of two autophagy-associated proteins. One is microtubule-associated protein 1 light chain 3 (LC3), a yeast Atg8 homolog, whose lipidated forms (LC3-II) are highly enriched onto autophagosomal membranes [32] (Figure 1). The other is p62 (a.k.a. sequestosome 1/SQSTM1), an adaptor molecule for selective autophagosomal degradation of ubiquitinated targets, which directly binds to LC3, thereby promoting the recruitment and engulfment of cargos to autophagosomes [33–35] (Figure 1).

### 2.2. Endocytic Pathways

Endocytosis is an evolutionally conserved cellular process involving the internalization of a wide variety of molecules from the surface of cells. There are at least four distinct well-recognized endocytic pathways in eukaryotes: phagocytosis, macropinocytosis, clathrin-mediated endocytosis (CME), and caveola-mediated endocytosis [36–38] (Figure 1). In addition, several other uncharacterized clathrin- and caveolin-independent pathways exist [38]. Each endocytic pathway mediates the transport of specific cargo molecules and delivers them to the correct destinations within cells. It is highly appreciated that most internalized vesicles and/or vacuoles containing specific cargos mature to or fuse with early endosomes before the cargos are delivered to their end destinations [38], and that a variety of the distinctive Rab GTPases, such as Rab5 and Rab7, control endocytosis and vesicle trafficking as well as cargo transportation [39]. Interestingly, a recent study has demonstrated that certain types of endocytosis (macropinocytosis) and autophagy are oppositely regulated by a phospholipid binding protein Annexin A5, suggesting a coordinated cross-talk between endocytic and autophagic pathways [40].

### 2.3. Maturation of Autophagosomes, Macropinosomes, and Endosomes

Nascent autophagosomes undergo a stepwise maturation, resulting in the creation of amphisomes and autolysosomes by fusion with multiple endocytic compartments, such as early endosomes, multivesicular bodies (MVBs), late endosomes, and lysosomes [41, 42]. Amphisomes, an intermediate hybrid vesicular compartment, contain both autophagosomal and endosomal contents, while autolysosomes are formed either from amphisomes or directly from autophagosomes by fusion with lysosomes [41] (Figure 1). Notably, not only the internalized vacuoles such as macropinosomes that are generated through macropinocytosis but also early endosomes themselves sequentially mature into MVBs, late endosomes, and lysosomes [15] (Figure 1). Further, it has recently been shown that fusion of autophagosomes with early endosomes is required for autophagy [43]. These findings strongly support an intimate crosstalk between autophagic and endocytic pathways particularly in their maturation steps.

Elaborate molecular mechanisms regulating the maturation of intracellular vesicular/vacuolar compartments have recently begun to emerge. Those include Rab-switching, phosphatidylinositol (PI) conversion, endosomal sorting complex required for transport (ESCRT) machinery, as well as lumenal acidification, as reviewed in detail elsewhere [15, 41, 42, 44]. Among the systems, the sequential action of the small GTPases Rab5 and Rab7, that is, the Ra5-Rab7 switching, plays a central role in the early step of the endosome maturation; early to late endosomes [15, 45, 46]. Further, it has been reported that the sequential reaction of Rab5-Rab21-Rab7 plays a pivotal role in macropinocytosis and the macropinosome maturation [47]. PI conversions mediated by VPS34 and PIKfyve also tightly link to the maturation from Rab5- to Rab7-positive endosomes [15]. Of particular, the Class III phosphatidylinositol-3 kinase (PI3K) complexes containing p150, Beclin-1, VPS34, and UVRAG positively regulate the maturation of both autophagosomes and endosomes [15, 42, 48]. On the other hand, the ESCRT complexes play a major role in the later step of the autophagosome, amphisome, and endosome maturation [49]. Moreover, histone deacetylase-6 (HDAC6), a ubiquitin-binding deacetylase acting as a central component of basal autophagy, selectively targets the ubiquitinated proteins to autophagosomes [35], and controls the autophagosome maturation rather than the autophagosome formation [50]. Despite of such recent progress, molecular mechanisms underlying coordinated regulation of multiple maturation steps by these factors are still incompletely understood.

Since autophagosomes as well as endosomes are motile within cells [51, 52], it is reasonable that their movements are linked to their maturation stages, particularly in differentiated neuronal cells. Indeed, Rab5 and Rab7 act in a coordinated manner in controlling the early stage of maturation and the axonal retrograde transport of vesicles in motor neurons [52]. Further, Snapin, a neuronal SNARE-binding protein acting as an adaptor linking late endosomes to the dynein complex, plays key roles not only in dynein-mediated retrograde transport but also in late endosomal-lysosomal maturation in neurons [53]. Most recently, it has also been shown that autophagosomes are formed and fuse with late endosomes and/or immature lysosomes distally, and their maturation progresses during transport along the axons in primary dorsal root ganglion (DRG) neurons [54].

### 2.4. Lysosomal Degradation

The final step of the autophagy-endolysosomal system is degradation of cargo molecules within lysosomes. Two classes of proteins; lysosomal acid hydrolases and lysosomal membrane proteins (LMPs), play essential roles in degradation of cargos in lysosomes. Lysosomal acid hydrolases such as cathepsins are involved not only in bulk degradation of substrates (cargos) but also in other physiological processes such as antigen processing. On the other hand, LMPs have a wide variety of functions including lumenal acidification, import of cytosolic proteins, and transport of degraded materials to cytosol. Excellent review article specialized on lysosome biogenesis is available elsewhere [55].

Lysosomal positioning is dynamically regulated by nutritional conditions, in which a starvation induces preferential relocalization of lysosomes from cell peripheries to the juxtanuclear regions close to the microtubule-organizing center (MTOC), thereby regulating the autophagic flux in cells [56]. In neurons, bidirectional movements of lysosomes within axons are observed [57, 58]. While autophagosomes and endosomes are also bidirectionally moved in the distal axons [54], they are rather exclusively transported in a retrograde direction upon fusion with lysosomal-associated membrane protein 1 (LAMP-1)-positive late endosomes and/or immature lysosomes [51, 52, 54, 57]. Further, fully matured lysosomes containing active lysosomal hydrolases are confined to the proximal region of axons or the cell body [54, 57]. Thus, autophagosomes and endosomes formed in axons must be transported to the cell body for a complete digestion of their cargos [27] (Figure 1). Recent evidence showing that defective Snapin-dynein-mediated retrograde transport in neurons results in the aberrant accumulation of immature lysosomes and impaired lysosomal degradation [53] supports this notion. Taken together, lysosomal degradation of either engulfed or internalized cargos in neurons might be strictly dependent on retrograde transport and late endosomal-lysosomal trafficking [51, 53, 58].

## 3. Dysfunction of the Autophagy-Endolysosomal System in Motor Neuron Diseases

Growing evidence supports a role of the autophagy-endolysosomal pathway in the pathogenesis of ALS/MNDs. Indeed, the accumulation of autophagosomes were observed in the spinal cord of sporadic ALS patients [59], indicating autophagic dysfunction in ALS. Autophagic dysfunction includes defects in the initiation (formation of autophagosomes) and/or maturation stages of autophagic processes, as well as imbalance between them, resulting in aberrant accumulation of misfolded and/or aggregated proteins within cells. Such pathological conditions disturb neuronal homeostasis, leading to neurodegeneration. In this section, we focus on causative and/or associated genes for ALS/MNDs, whose gene products functionally link to the autophagy-endolysosomal system; including *SOD1* [60], *FIG4* [61], *VCP* [62], *CHMP2B* [63], *SQSTM1* [64], *DCTN1* [65], *DYNC1H1* [66], and *RAB7A* [67] (Table 1). We describe *ALS2* [68, 69] and its product ALS2/alsin, an emerging regulator for autophagy-endolysosomal system [70, 71], in a separate section (see Section 4). Other ALS/MND causative genes, such as *TARDBP* [72], *OPTN* [73], and *UBQLN2* [74], which are also associated with protein degradation, are described in detail elsewhere [4, 75].

### 3.1. Superoxide Dismutase 1 (SOD1): ALS1

Mutations in *SOD1* that encodes superoxide dismutase 1 (SOD1) account for an approximately 20% of familial ALS cases [1]. It is currently believed that the SOD1-mediated dismutase enzymatic activity is not a major determinant for the phenotypic modification in ALS, since there is no correlation between disease severities and the SOD1 dismutase activities [76, 77]. Rather, the propensity for the aggregate formation associated with mutant SOD1 proteins, that is, gain of toxic function, might be related to the phenotypic expression of disease [78]. Recently, it has been reported that the normal as well as mutant SOD1 proteins are degraded both by the UPS and the autophagy-endolysosomal system [70, 79]. SOD1 mutants can be recognized by p62 in an ubiquitin-independent manner and targeted for degradation through the autophagy-endolysosomal pathway [80, 81]. Importantly, progressive enhancement of autophagy and/or decrease of autophagic flux are detected in a mutant SOD1 (SOD1^G93A^)-expressing ALS mouse model [82–84]. Most recently, heat-shook protein 70 (Hsp70) and Bcl2-associated athanogene 3 (BAG3) mediate the ubiquitination-independent autophagic degradation of misfolded proteins including SOD1 mutants [85]. It is also noted that SOD1 mutants directly bind to the retrograde motor protein complex, thereby disturbing axonal transport [86–88] (see Section 3.6). Taken together, it is conceivable that increased accumulation of SOD1 mutants as disease progresses disturb the autophagy-endolysosomal system.

### 3.2. Phospholipid Phosphatase Fig4: ALS11

**Figure 2:**
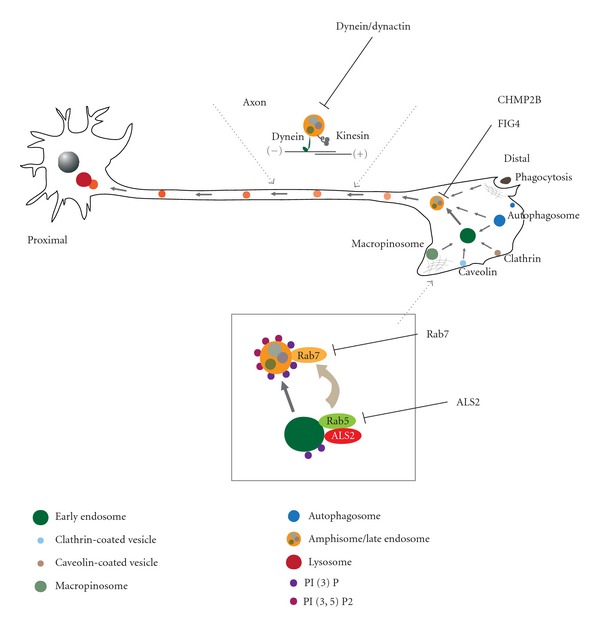
ALS-linked mutations in the genes, whose protein products are associated with autophagy-endolysosomal system and/or endocytic trafficking, underlie the pathogenesis of ALS and related motor neuron diseases.

Mutations in *FIG4* account for a form of autosomal recessive Charcot-Marie-Tooth type 4J (CMT4J) [89]. Interestingly, an approximately 2% of patients with ALS and primary lateral sclerosis (PLS) carry heterozygous deleterious mutations (nonsynonymous variants) in *FIG4* [61], indicating that *FIG4* is implicated in the pathogenesis of both peripheral neuropathy and ALS/MNDs. *FIG4* encodes a phosphoinositide 5-phosphatase, Fig4, that regulates the intracellular level of phosphatidylinositol-3,5,-bisphosphate (PI(3,5)P_2_). It has been shown that mutation in *FIG4* results in a significant reduction of the PI(3,5)P_2_ level in cultured cells [88]. Further, mice lacking Fig4, exhibit the accumulation of LC3-II, p62, and LAMP-2 in neurons and astrocytes, and die earlier than wild-type litters [90]. Thus, deregulation of the autophagy-endolysosomal system, namely the later stage of autophagosome and/or endosome maturation, might be associated with the pathogenesis of *FIG4*-linked ALS/MNDs (Figure 2).

### 3.3. Valosin-Containing Protein (VCP/p97): ALS14

Mutations in *VCP* have previously been identified in patients with inclusion body myopathy associated with Paget disease of bone and frontotemporal dementia (IBMPFD) [91]. Recently, exome sequencing reveals *VCP* mutations as a cause of familial ALS, accounting for 1-2% of familial ALS [62]. *VCP* encodes valosin-containing protein (VCP/p97) that belongs to the AAA+ (ATPases associated with various activities) protein family, being implicated in multiple cellular processes including the UPS [92, 93]. A recent study has shown that VCP/p97 regulates endolysosomal sorting of endocytosed ubiquitinated cargos such as caveolin-1 [94]. Further, loss of VCP/p97 accelerates the accumulation of autophagosomes [95], and expression of IBMPFD-linked mutants results in the impaired maturation of autolysosomes with accompanying the cytoplasmic accumulation of TAR DNA-binding protein (TDP-43), a causative gene product for ALS10 and a major constituent of ALS-linked cytoplasmic inclusions [72, 95–97]. Thus, VCP/p97 might play essential roles not only in the maturation of autophagosomes and endolysosomes, but also in the regulation of intracellular dynamics of TDP-43.

### 3.4. Charged Multivesicular Body Protein 2B (CHMP2B): ALS-FTD3

Mutations in *CHMP2B* have been identified in patients with FTD and ALS-FTD [63, 98]. *CHMP2B* encodes charged multivesicular body protein 2B (CHMP2B), a component of the ESCRT-III complex. The ESCRT complexes are known to play important roles in MVB biogenesis and autophagosomal-endolysosomal maturation [99]. Either functional loss of ESCRT-III or ectopic expression of disease-linked CHMP2B mutants causes the accumulation of LC3-positive autophagosomes accompanying protein aggregates containing ubiquitinated proteins and p62 [49], and results in dendritic retraction prior to neurodegeneration [100]. Interestingly, ESCRT-depleted cells also exhibit the accumulation of TDP-43 positive cytoplasmic inclusions [49]. These results indicate that deregulation of MVB biogenesis and autophagy is implicated in the pathogenesis of *CHMP2B*-linked FTD and ALS-FTD (Figure 2).

### 3.5. Sequestosome 1 (SQSTM1/p62)

*SQSTM1* encodes SQSTM1/p62 that was originally isolated as an interacting protein for the atypical protein kinases (aPKCs) [101]. It has been shown that p62 acts as an adaptor and/or scaffold protein that regulates not only the NF-*κ*B activation through the binding with aPKCs but also the selective-autophagy via association with ubiquitinated misfolded proteins [102–104]. Further, accumulation of p62 by defective autophagy causes competitive inhibition of the oxidative-stress responsive transcription factor Nrf2-Keap1 interaction, resulting in activation of Nrf2 and its target antioxidative stress genes [105]. Conversely, genetic inactivation of *Sqstm1 *in mice results in the accumulation of hyperphosphorylated tau and neurodegeneration [106]. Although mutations in *SQSTM1* have originally been identified in patients with Paget disease of bone (PDB) [107], a recent study has revealed several missense variants in *SQSTM1* in familial as well as sporadic ALS [64]. It is notable that abundant p62-positive inclusions in the brain are a typical pathological feature of ALS or ALS-FTD associated with hexanucleotide repeat expansion in *C9orf72* [108, 109]. Considering the facts that two independent genes linking to forms of Paget disease of bone; *VCP* for IBMPFD and *SQSTM1* for PDB, are also associated with ALS and/or ALS-FTD, and that both VCP and p62 are key regulators for the autophagy-endolysosomal system, dysregulation of such VCP/p62-associated common pathological pathways might account for these seemingly different diseases.

### 3.6. Dynein/Dynactin Complex

Mutation in* DCTN1 *encoding the p150 subunit of the transporter protein dynactin has been identified in autosomal dominant form of lower MNDs [65]. Dynactin functions as an adaptor between dynein and various cargos, thereby regulating the efficiency of dynein motor [11]. It has also been shown that expansion of polyglutamine-tract in androgen receptor, which causes a form of motor neuron disease; spinal and bulbar muscular atrophy (SBMA), results in polyglutamine-dependent transcriptional dysregulation of dynactin [110]. Moreover, overexpression of dynamitin (p50) subunit of dynactin, which causes a dissociation of the dynactin complex, thereby interfering the dynein/dynactin-dependent retrograde transport, causes MND in mice [111]. On the other hand, several studies identify mutations in the component of dynein motor complex itself. Exome sequencing reveals the mutation in *DYNC1H1 *encoding cytoplasmic dynein heavy chain in patients with dominant form of axonal CMT [66]. Mutations in a mouse homolog *Dync1h1 *have also been identified, resulting in progressive motor neuron degeneration in mice [86]. It has been demonstrated that a mutant SOD1-expressing ALS mouse model carrying dynein mutation shows a defective axonal transport [86, 87]. Further, SOD1 mutants preferentially interact with the dynein complex, disturbing their functions [88]. Interestingly, decreased dynein function impairs the autophagy-dependent clearance of misfolded protein aggregates in parallel with the increased level of LC3-II-positive autophagosomes [112]. Collectively, defects in dynein/dynactin-mediated retrograde axonal transport are involved in etiology of ALS/MNDs [11] (Figures 1 and 2).

### 3.7. Small GTPase Rab7

Charcot-Marie-Tooth type 2B (CMT2B) is an autosomal-dominant peripheral neuropathy caused by the missense mutations in *RAB7A* [67]. These mutations cause the constitutive activation of its encoding protein Rab7 [113], a regulator of maturation of autophagosomes, amphisomes, and late endosomes in cells [114–116] (Figures 1 and 2). Although the molecular mechanism by which dysfunction in a ubiquitously expressed Rab7 affects only sensory and/or motor neurons remains unclear, recent studies have demonstrated that these CMT2B-associated Rab7 mutants exhibit a persistent elevation of endosome-mediated nerve growth factor (NGF) signaling [117], and inhibit neurite outgrowth in cultured neuronal cells [118].

## 4. ALS2/alsin: A Regulator of Autophagy-Endolysosomal Protein Degradation

Loss of function mutations in the *ALS2* gene accounts for juvenile recessive amyotrophic lateral sclerosis (ALS2), juvenile primary lateral sclerosis (JPLS), and infantile-onset ascending hereditary spastic paralysis (IAHSP) [68, 69, 119, 120]. The *ALS2* gene encodes a 184 kDa protein of 1657 amino acids, ALS2 or alsin, comprising three predicted guanine nucleotide exchange factor (GEF) domains: the N-terminal RCC1-like domain (RLD), the central Dbl homology and pleckstrin homology (DH/PH) domain, and the C-terminal vacuolar protein sorting 9 (VPS9) domain [68]. Indeed, it has been shown that ALS2 acts as a GEF for Rab5 [120–122], and regulates endosome fusion and trafficking by activating Rab5 [120, 121, 123] (Figure 1). ALS2 is also involved in Rac1-activated macropinocytosis and the following macropinosome trafficking and fusion [124, 125]. In particular, fusion between early endosomes and macropinosomes is, at least in part, regulated by ALS2 in an ALS2-associated Rab5 GEF activity-dependent manner [124]. Further, ALS2 plays some modulatory roles in axonal outgrowth in neuronal cells [125, 126], and in cytoprotection from oxidative stress-induced insults [127–130].

 Recently, we have demonstrated that activated Rac1 interacts with ALS2 and induces the relocalization of ALS2 from cytoplasm to membranous compartments; for example, membrane ruffle, macropinosome, and endosome [124]. This Rac1-mediated relocalization of ALS2 is required for the ALS2-mediated Rab5 activation on the membranous compartments [71, 124]. It is noted that ALS2 is also colocalized with LC3/p62-positive autophagosomes and/or amphisomes [70, 71]. Conversely, pathogenic missense ALS2 mutants fail to be localized to such vesicular compartments, and lose the competence to enhance the formation of amphisomes [71], indicating that the Rac1-induced relocalization of ALS2 might be crucial to exert the ALS2-associated function linking to the autophagy-endolysosomal degradative pathway. Indeed, loss of ALS2 results in a slower degradation of endocytosed epidermal growth factor (EGF) in mouse embryonic fibroblasts [131]. Further, an ALS2-deficient SOD1^H46R^-expressing ALS mouse model exhibits the aberrant accumulation of autophagosomes and vesicular compartments in axons, delayed protein degradation by the autophagy-endolysosomal system, accelerated neurodegeneration, and earlier death [70]. Although the exact physiological function of ALS2 remains to be clarified, it is currently believed that ALS2 plays an important role in trafficking and maturation of several distinct vesicular compartments, including macropinosome, endosome, and autophagosome, and is implicated in the autophagy-endolysosomal degradative pathways (Figure 2).

## 5. Conclusions and Perspectives

Thus far, a large number of successful therapeutic interventions in preclinical animal studies have failed to translate into human clinical applications in ALS/MNDs. Even in such a discouraging situation, enormous efforts have continuously been made towards defining the molecular pathogenesis of these devastating diseases. The autophagy-endolysosomal system is among the underlying mechanisms, whose dysfunction is tightly associated with a variety of neurodegenerative diseases. It plays a pivotal role not only in ALS/MNDs as discussed in this paper, but also in other neurodegenerative diseases including Alzheimer's disease [57], Parkinson's disease [132], and Huntington's disease [133, 134]. Thus, the autophagy-endolysosomal pathway could be a major target for the development of novel therapeutic agents for neurodegenerative diseases [135]. Indeed, the induction of autophagy by lithium administration results in a reduced level of aggregated proteins and extends lifespan in a SOD1^G93A^-expressing ALS mouse model [136]. However, a recent preclinical animal study has demonstrated that the treatment with rapamycin, an another inducer of autophagy, rather causes the accumulation of p62, more severe mitochondrial impairment, higher Bax levels, and greater caspase-3 activation, thereby augmenting motor neuron degeneration in a same ALS mouse model [137]. These conflicting results imply that the simple pharmacological induction of autophagy cannot be always beneficial *in vivo*. As such, our understanding of the intricate autophagy-endolysosomal system and its functional linking to other physiological systems in the CNS is still incomplete. Future studies, which could uncover the molecular mechanisms of a selective neurodegeneration in greater detail, will be required for the development of proper and effective therapeutic agents for the treatment of ALS/MNDs and other neurodegenerative diseases.

## Figures and Tables

**Table 1:** Genes associated with ALS and other neurodegenerative diseases.

Disease type	Locus	Gene	Protein	Inheritance^∗^	Onset	Function	Mutation linked to other diseases
ALS1	21q22.11	*SOD1*	SOD1	D	Adult	Oxidative and ER stress response	
ALS2	2q33.1	*ALS2*	ALS2/alsin	R	Juvenile	Trafficking and protein degradation	PLSJ, IAHSP
ALS3	18q21	—	—	D	Adult	—	
ALS4	9q34.13	*SETX*	Senataxin	D	Juvenile	DNA damage response	AOA2
ALS5	15q21.1	*SPG11*	Spatacsin	R	Juvenile	—	SPG11
ALS6	16p11.2	*FUS*	FUS	D	Adult	DNA and RNA metabolism	ALS-FTD
ALS7	20p13	—	—	D	Adult	—	
ALS8	20q13.32	*VAPB*	VAPB	D	Adult	ER and Golgi membrane trafficking	SMA4
ALS9	14q11.2	*ANG*	Angiogenin	D	Adult	Neuroprotection	PD or ALS-PD
ALS10	1p36.22	*TARDBP*	TDP-43	D, R, or S	Adult	DNA and RNA metabolism	ALS-FTD, FTD
ALS11	6q21	*FIG4*	FIG4	D or S	Adult	PI (3,5) P2 regulation	CMT4J
ALS12	10p13	*OPTN*	Optineurin	D or R	Adult	NFkB regulation	GLC1E
ALS13	12q24.12	*ATXN2*	Ataxin-2	D	Adult	Gene regulation	SCA2
ALS14	9p13.3-p12	*VCP*	VCP or p97	D	Adult	Protein degradation	IBMPFD
ALS15	Xp11.21	*UNQLN2*	Ubiquilin-2	D	Adult	Protein degradation	ALS-FTD
ALS16	9p13.3	*SIGMAR1*	SIGMAR1	R	Juvenile	ER chaperon	
ALS-FTD1	9q21-q22	—	—	D or S	Adult	—	
ALS-FTD2	9p21.2	*C9orf72*	C9ORF72	D or S	Adult	—	FTD
ALS-FTD3	3p11.2	*CHMP2B*	CHMP2B	D	Adult	Trafficking and protein degradation	
DHN-7B	2p13.1	*DCTN1*	Dynactin-1	D	Adult	Trafficking	Perry syndrome
CMT2B	3q21.3	*RAB7*	Rab7	D	Adult	Trafficking and protein degradation	
CMT2O	14q32.31	*DYNC1H1*	Dynein	D	Adult	Trafficking	SMA-LED and MRD13
ALS^∗∗^	5q35.3	*SQSTM1*	Sequestosome or p62	?	Adult	Protein degradation	PDB

^∗^Inheritance (D: dominant, R: recessive, and S: sporadic). FTD: Frontotemporal dementia, DHN: distal hereditary motor neuronopathy, CMT: Charcot-Marie-Tooth disease, PDB: Paget disease of bone, PLSJ: primary lateral sclerosis juvenile, IAHSP: infantile-onset ascending hereditary spastic paralysis, AOA: ataxia-ocular apraxia-2, SPG: spastic paraplegia, SMA: spinal muscular atrophy, PD: Parkinson's disease, GLC1E: glaucoma 1, open angle, E, SCA2: spinocerebellar ataxia-2, IBMPFD: inclusion body myopathy with dementia and Paget disease of bone, SMA-LED: spinal muscular atrophy with lower limb predominance, and MRD13: mental retardation, autosomal dominant 13. ^∗∗^ALS: Fecoto et al. reported several novel SQSTM1 mutations in patients with ALS and predicted 8 of 9 missense variants behave like a pathogenic mutant by in silico analysis [64].

## References

[1] Andersen PM, Al-Chalabi A (2011). Clinical genetics of amyotrophic lateral sclerosis: what do we really know?. *Nature Reviews Neurology*.

[2] Hardiman O, van den Berg LH, Kiernan MC (2011). Clinical diagnosis and management of amyotrophic lateral sclerosis. *Nature Reviews Neurology*.

[3] Gordon PH, Meininger V (2011). How can we improve clinical trials in amyotrophic lateral sclerosis?. *Nature Reviews Neurology*.

[4] Ferraiuolo L, Kirby J, Grierson AJ, Sendtner M, Shaw PJ (2011). Molecular pathways of motor neuron injury in amyotrophic lateral sclerosis. *Nature Reviews Neurology*.

[5] Swarup V, Julien JP (2011). ALS pathogenesis: recent insights from genetics and mouse models. *Progress in Neuro-Psychopharmacology and Biological Psychiatry*.

[6] Pasinelli P, Brown RH (2006). Molecular biology of amyotrophic lateral sclerosis: insights from genetics. *Nature Reviews Neuroscience*.

[7] Barber SC, Mead RJ, Shaw PJ (2006). Oxidative stress in ALS: a mechanism of neurodegeneration and a therapeutic target. *Biochimica et Biophysica Acta*.

[8] Boillée S, Velde CV, Cleveland D (2006). ALS: a disease of motor neurons and their nonneuronal neighbors. *Neuron*.

[9] Kwak S, Weiss JH (2006). Calcium-permeable AMPA channels in neurodegenerative disease and ischemia. *Current Opinion in Neurobiology*.

[10] Nassif M, Matus S, Castillo K, Hetz C (2010). Amyotrophic lateral sclerosis pathogenesis: a journey through the secretory pathway. *Antioxidants and Redox Signaling*.

[11] Ström AL, Gal J, Shi P, Kasarskis EJ, Hayward LJ, Zhu H (2008). Retrograde axonal transport and motor neuron disease. *Journal of Neurochemistry*.

[12] Polymenidou M, Cleveland DW (2011). The seeds of neurodegeneration: prion-like spreading in ALS. *Cell*.

[13] Tai HC, Schuman EM (2008). Ubiquitin, the proteasome and protein degradation in neuronal function and dysfunction. *Nature Reviews Neuroscience*.

[14] Mizushima N (2007). Autophagy: process and function. *Genes and Development*.

[15] Huotari J, Helenius A (2011). Endosome maturation. *The EMBO Journal*.

[16] Agola J, Jim P, Ward H, Basuray S, Wandinger-Ness A Rab GTPases as regulators of endocytosis, targets of disease and therapeutic opportunities.

[17] Yue Z, Friedman L, Komatsu M, Tanaka K (2009). The cellular pathways of neuronal autophagy and their implication in neurodegenerative diseases. *Biochimica et Biophysica Acta*.

[18] Yamamoto A, Simonsen A (2011). The elimination of accumulated and aggregated proteins: a role for aggrephagy in neurodegeneration. *Neurobiology of Disease*.

[19] Bendotti C, Marino M, Cheroni C (2012). Dysfunction of constitutive and inducible ubiquitin-proteasome system in amyotrophic lateral sclerosis: implication for protein aggregation and immune response. *Progress in Neurobiology*.

[20] Levine B, Kroemer G (2008). Autophagy in the pathogenesis of disease. *Cell*.

[21] Longatti A, Tooze SA (2009). Vesicular trafficking and autophagosome formation. *Cell Death and Differentiation*.

[22] Mizushima N, Levine B, Cuervo AM, Klionsky DJ (2008). Autophagy fights disease through cellular self-digestion. *Nature*.

[23] Mizushima N, Komatsu M (2011). Autophagy: renovation of cells and tissues. *Cell*.

[24] Chua CEL, Gan BQ, Tang BL (2011). Involvement of members of the Rab family and related small GTPases in autophagosome formation and maturation. *Cellular and Molecular Life Sciences*.

[25] Nishida Y, Arakawa S, Fujitani K (2009). Discovery of Atg5/Atg7-independent alternative macroautophagy. *Nature*.

[26] He C, Bassik MC, Moresi V (2012). Exercise-induced BCL2-regulated autophagy is required for muscle glucose homeostasis. *Nature*.

[27] Komatsu M, Ueno T, Waguri S, Uchiyama Y, Kominami E, Tanaka K (2007). Constitutive autophagy: vital role in clearance of unfavorable proteins in neurons. *Cell Death and Differentiation*.

[28] Boland B, Kumar A, Lee S (2008). Autophagy induction and autophagosome clearance in neurons: relationship to autophagic pathology in Alzheimer’s disease. *Journal of Neuroscience*.

[29] Hara T, Nakamura K, Matsui M (2006). Suppression of basal autophagy in neural cells causes neurodegenerative disease in mice. *Nature*.

[30] Komatsu M, Waguri S, Chiba T (2006). Loss of autophagy in the central nervous system causes neurodegeneration in mice. *Nature*.

[31] Mizushima N, Yamamoto A, Matsui M, Yoshimori T, Ohsumi Y (2004). In vivo analysis of autophagy in response to nutrient starvation using transgenic mice expressing a fluorescent autophagosome marker. *Molecular Biology of the Cell*.

[32] Rubinsztein DC, Cuervo AM, Ravikumar B (2009). In search of an ‘autophagomometer’. *Autophagy*.

[33] Ichimura Y, Kumanomidou T, Sou YS (2008). Structural basis for sorting mechanism of p62 in selective autophagy. *Journal of Biological Chemistry*.

[34] Bjørkøy G, Lamark T, Pankiv S, Øvervatn A, Brech A, Johansen T (2009). Chapter 12 monitoring autophagic degradation of p62/SQSTM1. *Methods in Enzymology*.

[35] Kirkin V, McEwan DG, Novak I, Dikic I (2009). A role for ubiquitin in selective autophagy. *Molecular Cell*.

[36] Bowen S, Ateh DD, Deinhardt K (2007). The phagocytic capacity of neurones. *European Journal of Neuroscience*.

[37] McMahon HT, Boucrot E (2011). Molecular mechanism and physiological functions of clathrin-mediated endocytosis. *Nature Reviews Molecular Cell Biology*.

[38] Mayor S, Pagano RE (2007). Pathways of clathrin-independent endocytosis. *Nature Reviews Molecular Cell Biology*.

[39] Stenmark H (2009). Rab GTPases as coordinators of vesicle traffic. *Nature Reviews Molecular Cell Biology*.

[40] Ghislat G, Aguado C, Knecht E (2012). Annexin A5 stimulates autophagy and inhibits endocytosis. *Journal of Cell Science*.

[41] Fader CM, Colombo MI (2009). Autophagy and multivesicular bodies: two closely related partners. *Cell Death and Differentiation*.

[42] Simonsen A, Tooze SA (2009). Coordination of membrane events during autophagy by multiple class III PI3-kinase complexes. *The Journal of Cell Biology*.

[43] Razi M, Chan EYW, Tooze SA (2009). Early endosomes and endosomal coatomer are required for Autophagy. *The Journal of Cell Biology*.

[44] Noda T, Fujita N, Yoshimori T (2009). The late stages of autophagy: How does the end begin?. *Cell Death and Differentiation*.

[45] Rojas R, van Vlijmen T, Mardones GA (2008). Regulation of retromer recruitment to endosomes by sequential action of Rab5 and Rab7. *The Journal of Cell Biology*.

[46] Poteryaev D, Datta S, Ackema K, Zerial M, Spang A (2010). Identification of the switch in early-to-late endosome transition. *Cell*.

[47] Egami Y, Araki N (2009). Dynamic changes in the spatiotemporal localization of Rab21 in live RAW264 cells during macropinocytosis. *PLoS ONE*.

[48] Matsunaga K, Saitoh T, Tabata K (2009). Two beclin 1-binding proteins, Atg14L and Rubicon, reciprocally regulate autophagy at different stages. *Nature Cell Biology*.

[49] Filimonenko M, Stuffers S, Raiborg C (2007). Functional multivesicular bodies are required for autophagic clearance of protein aggregates associated with neurodegenerative disease. *The Journal of Cell Biology*.

[50] Lee JY, Koga H, Kawaguchi Y (2010). HDAC6 controls autophagosome maturation essential for ubiquitin-selective quality-control autophagy. *The EMBO Journal*.

[51] Katsumata K, Nishiyama J, Inoue T, Mizushima N, Takeda J, Yuzaki M (2010). Dynein- and activity-dependent retrograde transport of autophagosomes in neuronal axons. *Autophagy*.

[52] Deinhardt K, Salinas S, Verastegui C (2006). Rab5 and Rab7 control endocytic sorting along the axonal retrograde transport pathway. *Neuron*.

[53] Cai Q, Lu L, Tian JH, Zhu YB, Qiao H, Sheng ZH (2010). Snapin-regulated late endosomal transport is critical for efficient autophagy-lysosomal function in neurons. *Neuron*.

[54] Maday S, Wallace KE, Holzbaur ELF (2012). Autophagosomes initiate distally and mature during transport toward the cell soma in primary neurons. *The Journal of Cell Biology*.

[55] Saftig P, Klumperman J (2009). Lysosome biogenesis and lysosomal membrane proteins: trafficking meets function. *Nature Reviews Molecular Cell Biology*.

[56] Korolchuk VI, Saiki S, Lichtenberg M (2011). Lysosomal positioning coordinates cellular nutrient responses. *Nature Cell Biology*.

[57] Lee S, Sato Y, Nixon RA (2011). Lysosomal proteolysis inhibition selectively disrupts axonal transport of degradative organelles and causes an Alzheimer’s-like axonal dystrophy. *Journal of Neuroscience*.

[58] Yang Y, Feng LQ, Zheng XX (2011). Microtubule and kinesin/dynein-dependent, bi-directional transport of autolysosomes in neurites of PC12 cells. *International Journal of Biochemistry and Cell Biology*.

[59] Sasaki S (2011). Autophagy in spinal cord motor neurons in sporadic amyotrophic lateral sclerosis. *Journal of Neuropathology and Experimental Neurology*.

[60] Rosen DR, Siddique T, Patterson D (1993). Mutations in Cu/Zn superoxide dismutase gene are associated with familial amyotrophic lateral sclerosis. *Nature*.

[61] Chow CY, Landers JE, Bergren SK (2009). Deleterious variants of FIG4, a phosphoinositide phosphatase, in patients with ALS. *American Journal of Human Genetics*.

[62] Johnson JO, Mandrioli J, Benatar M (2010). Exome sequencing reveals VCP mutations as a cause of familial ALS. *Neuron*.

[63] Skibinski G, Parkinson NJ, Brown JM (2005). Mutations in the endosomal ESCRTIII-complex subunit CHMP2B in frontotemporal dementia. *Nature Genetics*.

[64] Fecto F, Yan J, Vemula SP (2011). SQSTM1 mutations in familial and sporadic amyotrophic lateral sclerosis. *Archives of Neurology*.

[65] Puls I, Jonnakuty C, LaMonte BH (2003). Mutant dynactin in motor neuron disease. *Nature Genetics*.

[66] Weedon M, Hastings R, Caswell R (2011). Exome sequencing identifies a DYNC1H1 mutation in a large pedigree with dominant axonal Charcot-Marie-Tooth disease. *American Journal of Human Genetics*.

[67] Verhoeven K, De Jonghe P, Coen K (2003). Mutations in the small GTP-ase late endosomal protein RAB7 cause Charcot-Marie-Tooth type 2B neuropathy. *American Journal of Human Genetics*.

[68] Hadano S, Hand CK, Osuga H (2001). A gene encoding a putative GTPase regulator is mutated in familial amyotrophic lateral sclerosis 2. *Nature Genetics*.

[69] Yang Y, Hentati A, Deng HX (2001). The gene encoding alsin, a protein with three guanine-nucleotide exchange factor domains, is mutated in a form of recessive amyotrophic lateral sclerosis. *Nature Genetics*.

[70] Hadano S, Otomo A, Kunita R (2010). Loss of ALS2/Alsin exacerbates motor dysfunction in a SOD1-expressing mouse ALS model by disturbing endolysosomal trafficking. *PloS ONE*.

[71] Otomo A, Kunita R, Suzuki-Utsunomiya K, Ikeda JE, Hadano S (2011). Defective relocalization of ALS2/alsin missense mutants to Rac1-induced macropinosomes accounts for loss of their cellular function and leads to disturbed amphisome formation. *FEBS Letters*.

[72] Neumann M, Sampathu DM, Kwong LK (2006). Ubiquitinated TDP-43 in frontotemporal lobar degeneration and amyotrophic lateral sclerosis. *Science*.

[73] Maruyama H, Morino H, Ito H (2010). Mutations of optineurin in amyotrophic lateral sclerosis. *Nature*.

[74] Deng H-X, Chen W, Hong S-T (2011). Mutations in UBQLN2 cause dominant X-linked juvenile and adult-onset ALS and ALS/dementia. *Nature*.

[75] Fecto F, Siddique T (2012). UBQLN2/P62 cellular recycling pathways in amyotrophic lateral sclerosis and frontotemporal dementia. *Muscle and Nerve*.

[76] Ratovitski T, Corson LB, Strain J (1999). Variation in the biochemical/biophysical properties of mutant superoxide dismutase 1 enzymes and the rate of disease progression in familial amyotrophic lateral sclerosis kindreds. *Human Molecular Genetics*.

[77] Reaume AG, Elliott JL, Hoffman EK (1996). Motor neurons in Cu/Zn superoxide dismutase-deficient mice develop normally but exhibit enhanced cell death after axonal injury. *Nature Genetics*.

[78] Bruijn LI, Houseweart MK, Kato S (1998). Aggregation and motor neuron toxicity of an ALS-linked SOD1 mutant independent from wild-type SOD1. *Science*.

[79] Kabuta T, Suzuki Y, Wada K (2006). Degradation of amyotrophic lateral sclerosis-linked mutant Cu,Zn-superoxide dismutase proteins by macroautophagy and the proteasome. *Journal of Biological Chemistry*.

[80] Gal J, Ström AL, Kilty R, Zhang F, Zhu H (2007). p62 accumulates and enhances aggregate formation in model systems of familial amyotrophic lateral sclerosis. *Journal of Biological Chemistry*.

[81] Gal J, Ström AL, Kwinter DM (2009). Sequestosome 1/p62 links familial ALS mutant SOD1 to LC3 via an ubiquitin-independent mechanism. *Journal of Neurochemistry*.

[82] Morimoto N, Nagai M, Ohta Y (2007). Increased autophagy in transgenic mice with a G93A mutant SOD1 gene. *Brain Research*.

[83] Li A, Zhang X, Le W (2008). Altered macroautophagy in the spinal cord of SOD1 mutant mice. *Autophagy*.

[84] Tian F, Morimoto N, Liu W (2011). In vivo optical imaging of motor neuron autophagy in a mouse model of amyotrophic lateral sclerosis. *Autophagy*.

[85] Gamerdinger M, Kaya AM, Wolfrum U, Clement AM, Behl C (2011). BAG3 mediates chaperone-based aggresome-targeting and selective autophagy of misfolded proteins. *EMBO Reports*.

[86] Hafezparast M, Klocke R, Ruhrberg C (2003). Mutations in dynein link motor neuron degeneration to defects in retrograde transport. *Science*.

[87] Ilieva HS, Yamanaka K, Malkmus S (2008). Mutant dynein (Loa) triggers proprioceptive axon loss that extends survival only in the SOD1 ALS model with highest motor neuron death. *Proceedings of the National Academy of Sciences of the United States of America*.

[88] Zhang F, Ström AL, Fukada K, Lee S, Hayward LJ, Zhu H (2007). Interaction between familial Amyotrophic Lateral Sclerosis (ALS)-linked SOD1 mutants and the dynein complex. *Journal of Biological Chemistry*.

[89] Chow CY, Zhang Y, Dowling JJ (2007). Mutation of FIG4 causes neurodegeneration in the pale tremor mouse and patients with CMT4J. *Nature*.

[90] Ferguson CJ, Lenk GM, Meisler MH (2009). Defective autophagy in neurons and astrocytes from mice deficient in PI(3,5)P2. *Human Molecular Genetics*.

[91] Watts GDJ, Wymer J, Kovach MJ (2004). Inclusion body myopathy associated with Paget disease of bone and frontotemporal dementia is caused by mutant valosin-containing protein. *Nature Genetics*.

[92] Yamanaka K, Sasagawa Y, Ogura T (2012). Recent advances in p97/VCP/Cdc48 cellular functions. *Biochimica et Biophysica Acta*.

[93] Meyer H, Bug M, Bremer S (2012). Emerging functions of the VCP/p97 AAA-ATPase in the ubiquitin system. *Nature Cell Biology*.

[94] Ritz D, Vuk M, Kirchner P (2011). Endolysosomal sorting of ubiquitylated caveolin-1 is regulated by VCP and UBXD1 and impaired by VCP disease mutations. *Nature Cell Biology*.

[95] Ju JS, Fuentealba RA, Miller SE (2009). Valosin-containing protein (VCP) is required for autophagy and is disrupted in VCP disease. *The Journal of Cell Biology*.

[96] Arai T, Hasegawa M, Akiyama H (2006). TDP-43 is a component of ubiquitin-positive tau-negative inclusions in frontotemporal lobar degeneration and amyotrophic lateral sclerosis. *Biochemical and Biophysical Research Communications*.

[97] Sreedharan J, Blair IP, Tripathi VB (2008). TDP-43 mutations in familial and sporadic amyotrophic lateral sclerosis. *Science*.

[98] Parkinson N, Ince PG, Smith MO (2006). ALS phenotypes with mutations in CHMP2B (charged multivesicular body protein 2B). *Neurology*.

[99] Henne WM, Buchkovich NJ, Emr SD (2011). The ESCRT pathway. *Developmental Cell*.

[100] Lee JA, Beigneux A, Ahmad ST, Young SG, Gao FB (2007). ESCRT-III dysfunction causes autophagosome accumulation and neurodegeneration. *Current Biology*.

[101] Sanchez P, De Carcer G, Sandoval IV, Moscat J, Diaz-Meco MT (1998). Localization of atypical protein kinase C isoforms into lysosome- targeted endosomes through interaction with p62. *Molecular and Cellular Biology*.

[102] Moscat J, Diaz-Meco MT (2009). p62 at the crossroads of autophagy, apoptosis, and cancer. *Cell*.

[103] Matsumoto G, Wada K, Okuno M, Kurosawa M, Nukina N (2011). Serine 403 phosphorylation of p62/SQSTM1 regulates selective autophagic clearance of ubiquitinated proteins. *Molecular Cell*.

[104] Komatsu M, Ichimura Y (2010). Physiological significance of selective degradation of p62 by autophagy. *FEBS Letters*.

[105] Komatsu M, Kurokawa H, Waguri S (2010). The selective autophagy substrate p62 activates the stress responsive transcription factor Nrf2 through inactivation of Keap1. *Nature Cell Biology*.

[106] Ramesh Babu J, Lamar Seibenhener M, Peng J (2008). Genetic inactivation of p62 leads to accumulation of hyperphosphorylated tau and neurodegeneration. *Journal of Neurochemistry*.

[107] Laurin N, Brown JP, Morissette J, Raymond V (2002). Recurrent mutation of the gene encoding sequestosome 1 (SQSTM1/p62) in paget disease of bone. *American Journal of Human Genetics*.

[108] Troakes C, Maekawa S, Wijesekera L An MND/ALS phenotype associated with C9orf72 repeat expansion: abundant p62-positive, TDP-43-negative inclusions in cerebral cortex, hippocampus and cerebellum but without associated cognitive decline.

[109] Al-Sarraj S, King A, Troakes C (2011). P62 positive, TDP-43 negative, neuronal cytoplasmic and intranuclear inclusions in the cerebellum and hippocampus define the pathology of C9orf72-linked FTLD and MND/ALS. *Acta Neuropathologica*.

[110] Katsuno M, Adachi H, Minamiyama M (2006). Reversible disruption of dynactin 1-mediated retrograde axonal transport in polyglutamine-induced motor neuron degeneration. *Journal of Neuroscience*.

[111] LaMonte BH, Wallace KE, Holloway BA (2002). Disruption of dynein/dynactin inhibits axonal transport in motor neurons causing late-onset progressive degeneration. *Neuron*.

[112] Ravikumar B, Acevedo-Arozena A, Imarisio S (2005). Dynein mutations impair autophagic clearance of aggregate-prone proteins. *Nature Genetics*.

[113] Spinosa MR, Progida C, De Luca A, Colucci AMR, Alifano P, Bucci C (2008). Functional characterization of Rab7 mutant proteins associated with Charcot-Marie-Tooth type 2B disease. *Journal of Neuroscience*.

[114] Jäger S, Bucci C, Tanida I (2004). Role for Rab7 in maturation of late autophagic vacuoles. *Journal of Cell Science*.

[115] Bains M, Zaegel V, Mize-Berge J, Heidenreich KA (2011). IGF-I stimulates Rab7-RILP interaction during neuronal autophagy. *Neuroscience Letters*.

[116] Lin MG, Zhong Q (2011). Interaction between small GTPase Rab7 and PI3KC3 links autophagy and endocytosis a new Rab7 effector protein sheds light on membrane trafficking pathways. *Small Gtpases*.

[117] Basuray S, Mukherjee S, Romero E, Wilson MC, Wandinger-Ness A (2010). Rab7 mutants associated with charcot-Marie-Tooth disease exhibit enhanced NGF-stimulated signaling. *PLoS ONE*.

[118] Cogli L, Progida C, Lecci R, Bramato R, Krüttgen A, Bucci C (2010). CMT2B-associated Rab7 mutants inhibit neurite outgrowth. *Acta Neuropathologica*.

[119] Eymard-Pierre E, Lesca G, Dollet S (2002). Infantile-onset ascending hereditary spastic paralysis is associated with mutations in the alsin gene. *American Journal of Human Genetics*.

[120] Hadano S, Kunita R, Otomo A, Suzuki-Utsunomiya K, Ikeda JE (2007). Molecular and cellular function of ALS2/alsin: implication of membrane dynamics in neuronal development and degeneration. *Neurochemistry International*.

[121] Otomo A, Hadano S, Okada T (2003). ALS2, a novel guanine nucleotide exchange factor for the small GTPase Rab5, is implicated in endosomal dynamics. *Human Molecular Genetics*.

[122] Topp JD, Gray NW, Gerard RD, Horazdovsky BF (2004). Alsin is a Rab5 and Rac1 guanine nucleotide exchange factor. *Journal of Biological Chemistry*.

[123] Kunita R, Otomo A, Mizumura H (2004). Homo-oligomerization of ALS2 through its unique carboxyl-terminal regions is essential for the ALS2-associated Rab5 guanine nucleotide exchange activity and its regulatory function on endosome trafficking. *Journal of Biological Chemistry*.

[124] Kunita R, Otomo A, Mizumura H, Suzuki-Utsunomiya K, Hadano S, Ikeda JE (2007). The Rab5 activator ALS2/alsin acts as a novel Rac1 effector through Rac1-activated endocytosis. *Journal of Biological Chemistry*.

[125] Otomo A, Kunita R, Suzuki-Utsunomiya K (2008). ALS2/alsin deficiency in neurons leads to mild defects in macropinocytosis and axonal growth. *Biochemical and Biophysical Research Communications*.

[126] Jacquier A, Buhler E, Schäfer MKE (2006). Alsin/Rac1 signaling controls survival and growth of spinal motoneurons. *Annals of Neurology*.

[127] Cai H, Lin X, Xie C (2005). Loss of ALS2 function is insufficient to trigger motor neuron degeneration in knock-out mice but predisposes neurons to oxidative stress. *Journal of Neuroscience*.

[128] Taylor RC, Acquaah-Mensah G, Singhal M, Malhotra D, Biswal S (2008). Network inference algorithms elucidate Nrf2 regulation of mouse lung oxidative stress. *PLoS Computational Biology*.

[129] Li Q, Spencer NY, Pantazis NJ, Engelhardt JF (2011). Alsin and SOD1 (G93A) proteins regulate endosomal reactive oxygen species production by glial cells and proinflammatory pathways responsible for neurotoxicity. *Journal of Biological Chemistry*.

[130] Carter BJ, Anklesaria P, Choi S, Engelhardt JF (2009). Redox modifier genes and pathways in amyotrophic lateral sclerosis. *Antioxidants and Redox Signaling*.

[131] Hadano S, Benn SC, Kakuta S (2006). Mice deficient in the Rab5 guanine nucleotide exchange factor ALS2/alsin exhibit age-dependent neurological deficits and altered endosome trafficking. *Human Molecular Genetics*.

[132] Youle RJ, Narendra DP (2011). Mechanisms of mitophagy. *Nature Reviews Molecular Cell Biology*.

[133] Ravikumar B, Imarisio S, Sarkar S, O’Kane CJ, Rubinsztein DC (2008). Rab5 modulates aggregation and toxicity of mutant huntingtin through macroautophagy in cell and fly models of Huntington disease. *Journal of Cell Science*.

[134] Sarkar S, Krishna G, Imarisio S, Saiki S, O’Kane CJ, Rubinsztein DC (2008). A rational mechanism for combination treatment of Huntington’s disease using lithium and rapamycin. *Human Molecular Genetics*.

[135] Fleming A, Noda T, Yoshimori T, Rubinsztein DC (2011). Chemical modulators of autophagy as biological probes and potential therapeutics. *Nature Chemical Biology*.

[136] Fornai F, Longone P, Cafaro L (2008). Lithium delays progression of amyotrophic lateral sclerosis. *Proceedings of the National Academy of Sciences of the United States of America*.

[137] Zhang X, Li L, Chen S (2011). Rapamycin treatment augments motor neuron degeneration in SOD1^G93A^ mouse model of amyotrophic lateral sclerosis. *Autophagy*.

